# Changes in Functional Status Among Clusters of Older Adults After Hospitalization for Pneumonia

**DOI:** 10.1093/geroni/igab046.2281

**Published:** 2021-12-17

**Authors:** Chan Mi Park, Hye Chang Rhim, Eun Sik Lee, Wonsock Kim, Jong Hun Kim, Dae Hyun Kim

**Affiliations:** 1 Harvard T.H.Chan School of Public Health , Brookline, United States; 2 Harvard University, Orlando, Florida, United States; 3 Korea University Anam Hospital, Korea University Anam Hospital, Seoul-t'ukpyolsi, Republic of Korea; 4 Eulji Medical Center, Eulji Medical Center, Kyonggi-do, Republic of Korea; 5 CHA Bundang Medical Center, CHA Bundang Medical Center, Kyonggi-do, Republic of Korea; 6 Hebrew SeniorLife, roslindale, Massachusetts, United States

## Abstract

Little is known about how social determinants, comorbidity, and disability status are associated with functional recovery after an acute illness. A prospective cohort study was conducted between 2019-2020 at a university hospital in Korea, to investigate functional recovery after hospitalization for pneumonia in older adults with different degrees of social deprivation, disabilities, and comorbidities. K-means cluster analysis was used to identify groups of patients based on social deprivation score, activities of daily living, instrumental activities of daily living, physical limitation score, and Gagne comorbidity index. Four groups were identified: Group A: non-disabled group with limited social support (n=61 [30.3%]); Group B: multimorbid but non-disabled group with social support (n=45 [22.4%]); Group C: multimorbid and disabled group with social support (n=38 [18.9%]); Group D: multimorbid and disabled group with limited social support (n=57 [28.4%]). Functional status, defined as ability to perform 21 activities and physical tasks independently, was measured via telephone interviews at 1, 3, and 6 months after discharge. Group-based trajectory model identified four functional status trajectories: excellent (n=29 [14.4%]), good (n=51 [25.4%]), fair (n=58 [28.9%]) and poor (n=63 [31.3%]). The most common functional trajectory by four groups was good trajectory (59%) in Group A, excellent trajectory (48.9%) in Group B, fair (50%) and poor trajectory (50%) in Group C, and poor trajectory (77.2%) in Group D. Our results suggest that most patients without disability recover functional status after pneumonia, despite multimorbidity or limited social support. Social support seems to be more important for those with multimorbidity and disability.

